# A unified classification system for HIV-1 5’ long terminal repeats

**DOI:** 10.1371/journal.pone.0301809

**Published:** 2024-05-02

**Authors:** Xing Guo, Dan Yu, Mengying Liu, Hanping Li, Mingyue Chen, Xinyu Wang, Xiuli Zhai, Bohan Zhang, Yanglan Wang, Caiqing Yang, Chunlei Wang, Yongjian Liu, Jingwan Han, Xiaolin Wang, Jingyun Li, Lei Jia, Lin Li

**Affiliations:** 1 Department of Microbiology, School of Basic Medicine, Anhui Medical University, Hefei, Anhui, China; 2 Department of Virology, Beijing Institute of Microbiology and Epidemiology, Beijing, China; 3 State Key Laboratory of Pathogen and Biosecurity, Beijing, China; 4 Laboratory of Dermatology, Key Laboratory of Major Diseases in Children, Ministry of Education, Beijing Pediatric Research Institute, Beijing Children’s Hospital, National Center for Children’s Health, Capital Medical University, Beijing, China; 5 College of Life Science and Technology, Beijing University of Chemical Technology, Beijing, China; 6 National 111 Center for Cellular Regulation and Molecular Pharmaceutics, Key Laboratory of Fermentation Engineering, Hubei University of Technology, Wuhan, Hubei, China; University of Verona, ITALY

## Abstract

The HIV-1 provirus mainly consists of internal coding region flanked by 1 long terminal repeats (LTRs) at each terminus. The LTRs play important roles in HIV-1 reverse transcription, integration, and transcription. However, despite of the significant study advances of the internal coding regions of HIV-1 by using definite reference classification, there are no systematic and phylogenetic classifications for HIV-1 5’ LTRs, which hinders our elaboration on 5’ LTR and a better understanding of the viral origin, spread and therapy. Here, by analyzing all available resources of 5’ LTR sequences in public databases following 4 recognized principles for the reference classification, 83 representatives and 14 consensus sequences were identified as representatives of 2 groups, 6 subtypes, 6 sub-subtypes, and 9 CRFs. To test the reliability of the supplemented classification system, the constructed references were applied to identify the 5’ LTR assignment of the 22 clinical isolates in China. The results revealed that 16 out of 22 tested strains showed a consistent subtype classification with the previous LTR-independent classification system. However, 6 strains, for which recombination events within 5’ LTR were demonstrated, unexpectedly showed a different subtype classification, leading a significant change of binding sites for important transcription factors including SP1, p53, and NF-κB. The binding change of these transcriptional factors would probably affect the transcriptional activity of 5’ LTR. This study supplemented a unified classification system for HIV-1 5’ LTRs, which will facilitate HIV-1 characterization and be helpful for both basic and clinical research fields.

## Introduction

Human immunodeficiency virus type 1 (HIV-1) is the most widespread type of HIV and has many subtypes, which have caused a complex problem for vaccine design and development. HIV-1 provirus contains 1 long terminal repeats (LTRs) at each terminus after integration. Each flanking viral LTR consists of U3 (455bp), R (96 bp), and U5 (83bp) [1, 2], contains elements of promoters, enhancers, and multiple transcription factor binding sites (TFBS) [3–5], and plays an important role in virus reverse transcription [6], integration [7–11], and transcription [5, 12, 13]. There are four main regions according to function, relative to the the transcription start site (TSS) at +1 in HIV-1 LTR: the modulatory region (−455 to −104), the enhancer element (−109 to −79), the core promoter (−78 to −1), and the TAR region (+1 to +60). The region downstream of the TSS (+60 to +278) also binds TFs which play important roles in the transcriptional modulation [5]. Although both 5’ and 3’ LTRs have the function of Tat-induced promoter, the 5’ LTR element was the main transcriptional promoter, due to the fact that only the inactivation of a 5’ LTR promoter or lack of transcriptional function can trigger 3’ LTR transcription [12]. A large number of transcription factors (TFs) have been shown to bind HIV-1 5’ LTR and regulate proviral transcription [5], such as nuclear factor- κB (NF-κB), nuclear factor for activated T cells (NFAT), specific protein 1 (SP1), TATA-binding protein (TBP), activator protein-1 (AP-1) [13, 14] and the positive transcriptional elongation factor b (P-TEFb) [15, 16].

Since the discovery of HIV-1, reference sequences for HIV-1 internal region, 5’ LTR, or the full length has been proposed sporadically [2, 17–21]. However, the unified reference guide to HIV-1 classification based on phylogenetics was systematically proposed by Robertson, D., et al., till 2000 by using the internal regions including *gag*, *pol*, *env*, and other 6 function regions [22]. Taking advantage of these achievements, researchers have demonstrated that HIV-1 exhibit significant genetic diversity [6, 22, 23]. Currently, HIV-1 has been divided into 4 groups including Main (M), Outlier (O), non-M/non-O (N), and P groups. M group is the most circulating group, which has been divided into 10 subtypes and 9 sub-subtypes. In addition, there are 134 circulating recombinant forms (CRFs) and numberless unique recombinant forms (URFs).

However, the classification system mentioned above used the internal regions for HIV-1 classification and not 5’ HIV-1 LTRs. Despite of the significant study advances of the internal regions of HIV-1 by using definite reference classification, there are no systematic phylogenetics classifications for HIV-1 5’ LTRs, which hinders our elaboration on 5’ LTR and a better understanding of the viral origin and spread [2, 17–19, 24]. Intersubtype recombination in HIV-1 LTR has been demonstrated as an important viral evolutionary strategy, which also can affect the propagation of the virus [25, 26]. However, current analysis tools such as the posterior probabilities-based HIV-1 recombination detection tool, jpHMM, are unable to provide accurate subtyping information of the 5’ LTR region [27, 28]. Besides, the cis-acting elements and the various motifs of 5’ LTR have been widely studied for HIV-1 subtype B, but only a few works have focused on the other HIV-1 subtypes [16, 18, 24, 29] due to the unavailability of 5’ LTRs classification.

With the increasing recognition of the important roles played by 5’ LTR, a systematic and phylogenetic classification of 5’ LTR regions is needed. Therefore, we have supplemented a unified classification system for HIV-1 5’ LTR and found that a considerable proportion of strains from patients samples showed distinct subtype assignment by using our system in comparison with the previous LTR-independent classification system, highlighting a critical role of LTR references for HIV-1 classification. This classification system will help to improve our research on HIV-1 epidemiology and evolution, and better understand the basic and clinical science fields of HIV-1.

## Materials and methods

### Sequences

All available 5’ LTR sequences of HIV-1 (any subtype) were retrieved from the HIV sequence database on August 17, 2021, including solo 5’ LTRs without the remaining complete genome and those 5’ LTRs with the remaining complete genome. The box of *one sequence/patient* was checked. The term of *genomic regions* in the HIV sequence database includes 5’ LTR as the representative of HIV-1 LTRs (https://www.hiv.lanl.gov/components/sequence/HIV/search/search.html). The sequence materials of the current work were mainly retrieved from the HIV sequence database. Therefore, 5’ LTR is used in the title and text to be consistent with the current customary practice.

### Quality control

Multiple quality control strategies were performed to screen credible ones for reference construction and further analysis. Full-genome sequences assigned as unique recombinant form (URF) in the HIV sequence database were first excluded. The rest were submitted to the QC tool (https://www.hiv.lanl.gov/content/sequence/QC/index.h-tml) and jpHMM (http://jphmm.gobics.de/) to validate the subtype assignment and perform further elimination. JpHMM tool is based on posterior probabilities and is rather intelligent. It can produce a genome subtype mosaic map. The recombination prediction in jpHMM is based on a precalculated multiple sequence alignment of the major HIV-1 subtypes references. The evaluation of its prediction accuracy showed that it is more accurate than the competing methods used for recombination breakpoint detection [27, 28, 30]. To ensure that the most accurate sequences can be screened for reference construction, those full-genome sequences with inconsistent subtype information were also excluded. Similarly, solo 5’ LTRs assigned as URF or without subtype information were excluded. The length of all the remaining sequences was further examined by comparison to reference HXB2 (accession number: K03455) to exclude sequences exceeding 110% or less than 90% of the reference sequence length. Finally, the screened high-quality sequences were preserved for subsequent comprehensive classification.

### The 5’ LTR determination of HIV-1 strains circulating in China

Here, plasma samples were collected from HIV-1-infected patients from March 2020 to January 2021 in Hebei province, China. These plasma samples were stored at -80°C until use. Viral RNA was extracted using QIAamp^®^ Viral RNA Mini Kit (QIAGEN, Germany, 52904) according to the manufacturer’s instructions. Each flanking viral LTR of a HIV-1 provirus consists of U3 (455bp), R (96 bp), and U5 (83bp) [1, 2], and 5’ LTR is exactly the same to 3’ LTR. However, the genomic RNA (gRNA) of a progeny viruse only has R-U5 at 5’ LTR and U3-R at 3’ LTR. Thus, during the reverse transcription, a very smart replication strategy is used to generate a complete LTR in the provirus. During process of HIV-1 reverse transcription, R-U5 at 5’ LTR is first replicated, after unmasking of the nascent minus-strand strong-stop DNA by RNase-H degradation of the template RNA strand, the replicative template switch occurs for complementary R-region sequences near the 3’ LTR of the gRNA, and minus-strand DNA synthesis continues to form a complete LTR [6]. Exactly following the natural reverse transcription process of HIV-1, the two partial LTRs at the 5’ terminus and 3’ terminus of genomic RNA were amplified, respectively, and then sequenced, assembled, and combined via the R region for a complete LTR [6, 31, 32]. The primers in the non-coding regions (NCR) region being located at the junction of 5’ LTR and *gag* from 635–789 bp according to the coordinate of the HXB2 genome, and U3-R were designed. The nested PCR primers and their relative positions to reference HXB2 were shown in S1 Table. The reverse transcription and the first round PCR were performed in a 25μl reaction volume by using PrimeScript™ One-Step RT-PCR Kit (TAKARA, RR055A). Cycling conditions were as follows: initial incubation at 50°C for 32 min, and at 94°C for another 3 min. Subsequently, 30 cycles were performed at 94°C for 30 sec, at 55°C for 30 sec, and at 72°C for 1 min followed by a final extension of 5 min at 72°C. We performed the second round of PCR using Premix Taq™ (TAKARA, RR902Q) in a 50μl reaction volume. Cycling conditions were as follows: initial incubation at 94°C for 3 min followed by 35 cycles at 94°C for 30 sec, at 60°C for 30 sec, and at 72°C for 1 min, a final extension at 72°C for 10 min. The PCR products were delivered to Beijing Nuosai Genome Research Center (SinoGenoMax) Co., Ltd. under 0°C to 4°Cconditions. The sequencing was performed using Sanger method. The primary sequences were assembled and edited by CONTING EXPRESS.

### Phylogenetic analysis

To confirm 5’ LTR references classification, the screened solo 5’ LTR sequences and 5’ LTRs with complete sequences were used to construct a maximum likelihood phylogenetic tree with MEGA X software, respectively [33]. A maximum likelihood phylogenetic tree was also constructed to identify clinical isolates in Hebei province, China in the same way. The best-fitting models of nucleotide substitution were calculated by the model selection function in MEGA X. Tree topologies were searched using subtree-pruning-and-regrafting level 3 (SPR level 3), and the initial tree was made automatically (Default-NJ/BioNJ). The confidence of each node in phylogenetic trees was determined using the bootstrap test with 500 replicates. Bootstrap support values of ≥70% were considered to be significant. The final ML trees were visualized using iTOL v6 [34].

### Recombination analysis

There are 4 recognized requirements for subtype reference classification: (1) one or more complete sequence(s); (2) at least three epidemiologically unrelated isolates; (3) distinct clusters in a phylogenetic tree; (4) the exclusion of intersubtypic recombination, whether the components were classified or not [22, 35]. The reason for the 4^th^ is that compared to nucleotide substitution, recombination can dramatically change the content of genome and confuse the phylogenetic relationship [36, 37]. To investigate whether the recombination events exist among the potential LTR references and the clinical sequences, RDP4 recombination analysis tool was used to perform systematic recombination analysis [38]. RDP4 is a software package suitable for the statistical identification and characterization of recombination events in nucleotide sequences. It is very intelligent and can greatly improve both the sensitivity and reliability by using a series of nonparametric recombination detection methods: RDP, GENECONV, BOOTSCAN, MAXCHI, CHIMERA, SISCAN, 3SEQ, and LARD [39–46]. Here, the highest acceptable *p*-value is set to 0.05. The sequence is set to linear. Other parameters are the default RDP4 settings. In order to ensure reliability, an HIV-1 5’ LTR sequence is considered to be recombinant when the recombinant signal is supported by at least four methods with the *p*-value≤0.05 after Bonferroni correction. The inferred breakpoint position is manually checked using the recombinant signal analysis implemented in RDP4. For details on the methods and algorithms of the recombination analysis tools used here, please refer to the comprehensive list of recombination analysis softwares maintained by Robertson laboratory [47].

### Specific TFBS identification in 5’ LTRs

The PROMO tool was used to find the characteristic TFBS in different subtypes and recombinants within 5’ LTRs. PROMO is a virtual laboratory for the identification of putative TFBS in DNA sequences from a species or groups of species of interest. TFBS defined in the TRANSFAC database are used to construct specific binding site weight matrices for TFBS prediction [48, 49].

### Ethics statement

The study involving human participants was reviewed and approved by the Ethics Committee of STD AIDS Prevention and Control Center, Chinese Center for Disease Control and Prevention (X190311577). All participants signed written informed consent prior to sample donations.

## Results

### Sequences

**Fig 1:**
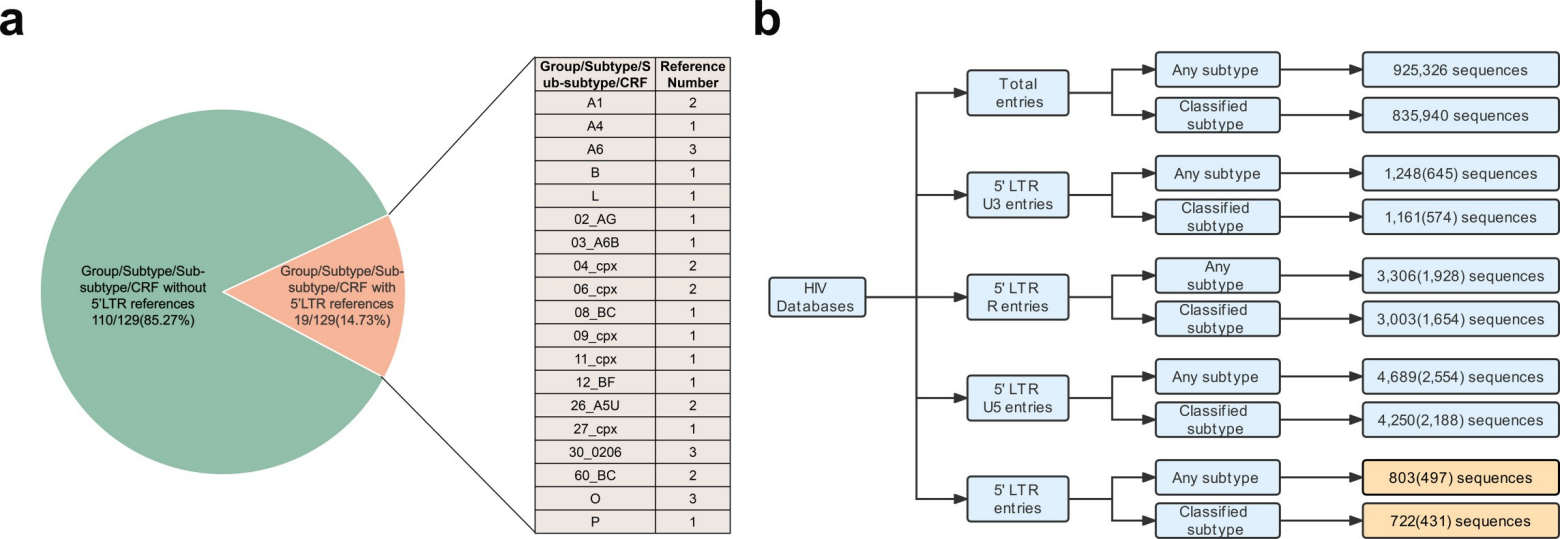
Inclusion of 5’ LTR region in HIV sequence database. (a) Completeness of 5’ LTR region in recognized *Subtype Reference Alignments* provided in the HIV sequence database. By August 17, 2021, among 129 the currently identified groups, subtypes, sub-subtypes, and CRFs representatives, only A1, A4, A6, B, L, CRF02_AG, CRF03_A6B, CRF04_cpx, CRF06_cpx, CRF08_BC, CRF09_cpx, CRF11_cpx, CRF12_BF, CRF26_A5U, CRF27_cpx, CRF30_0206, CRF60_BC, O, and P have 5’ LTR region. (b) Entries summary of HIV-1 sequences in the Los Alamos National Laboratory HIV sequence database by August 17, 2021.

There are currently 129 representatives for identified groups, subtypes, sub-subtypes, and CRFs in *Subtype Reference Alignments* (https://www.hiv.lanl.gov/content/sequence/NEWALIGN/help.html#ref) provided in the HIV sequence database. They are widely recognized and used in the field, and therefore are named gold-standard sequences in the current work. Among them, A1, A4, A6, B, L, CRF02_AG, CRF03_A6B, CRF04_cpx, CRF06_cpx, CRF08_BC, CRF09_cpx, CRF11_cpx, CRF12_BF, CRF26_A5U, CRF27_cpx, CRF30_0206, CRF60_BC, O, and P subtypes contain the sequence of 5’ LTR. All of the remaining 110 representatives have no sequence of 5’ LTR, accounting for 85.27% (110/129) (Fig 1A). Additionally, there are a total of 835,940 entries with classified subtypes by August 17, 2021. There are 803 5’ LTR entries by searching with any subtype, among which there are only 497 5’ LTR entries (one per patient) (Fig 1B). There are 722 5’ LTR entries by searching with classified subtype term, among which there are only 431 5’ LTR entries (one per patient) (Fig 1B). Then, a total of 497 5’ LTR sequences (any subtype) of HIV-1 were finally retrieved from HIV sequence database (S1 Dataset), which were divided into two subsets. One subset included 411 5’ LTR sequences, which contained the remaining genome region of HIV-1. Another subset included 86 5’ LTR sequences with no region of the remaining HIV-1 genome, which were defined as solo 5’ LTR.

### Quality control results

To improve the reliability of the reference classification, multiple quality control strategies have been used. Defective sequences with low quality were excluded. As for full-genome sequences, 44 out of 411 were first excluded for being assigned as URFs in the HIV sequence database. The remaining 367 full-length sequences were submitted to the Quality Control tool and jpHMM to further validate assignment accuracy. A total of 36 sequences were removed for no feedback by jpHMM. A total of 26 sequences were eliminated for subtype inconsistency. 3 sequences belonging to uncertain subtype definitions were also excluded. 27 sequences belonging to group O and 1 sequence belonging to group P were preserved as outgroup. As for solo 5’ LTRs, 5 sequences assigned as URFs in the HIV sequence database were excluded and another 2 solo 5’ LTRs were excluded for no subtype information. 1 sequence belonging to U (unkonwn) was excluded. Subsequently, the length of all sequences was further examined by comparison to reference HXB2 (accession number: K03455). Another 2 solo 5’ LTRs were excluded due to excessive length (2185 bp and 1920 bp) resulting from insertions. The details of excluded sequences are summarized in S2 Table. Finally, a total of 302 5’ LTRs with full-length sequences and 76 solo 5’ LTR sequences without complete genome were screened for further study (S2 and S3 Datasets).

### Provisional 5’ LTR reference classification from gold-standard sequences

There are 4 recognized requirements for subtype references classification: (1) one or more complete sequence(s); (2) at least three epidemiologically unrelated isolates; (3) distinct clusters in a phylogenetic tree; (4) exclusion of intersubtypic recombination, whether the components were classified or not [22, 35, 50]. Because sequences included in *Subtype Reference Alignments* have been strictly screened and recognized as standard references and widely used in related research of the field [51–53], they were named gold-standard sequences in this study. We found that among the 302 screened 5’ LTRs with full-length sequences, 20 sequences belong to gold-standard sequences (S4 Dataset). 5’ LTRs of these 20 gold-standard sequences have great potential to become high-quality reference sequences of the corresponding subtype and thus were provisionally assigned as the references for each subtype (S3 Table).

### Reference classification based on solo 5’ LTRs sequences

**Fig 2:**
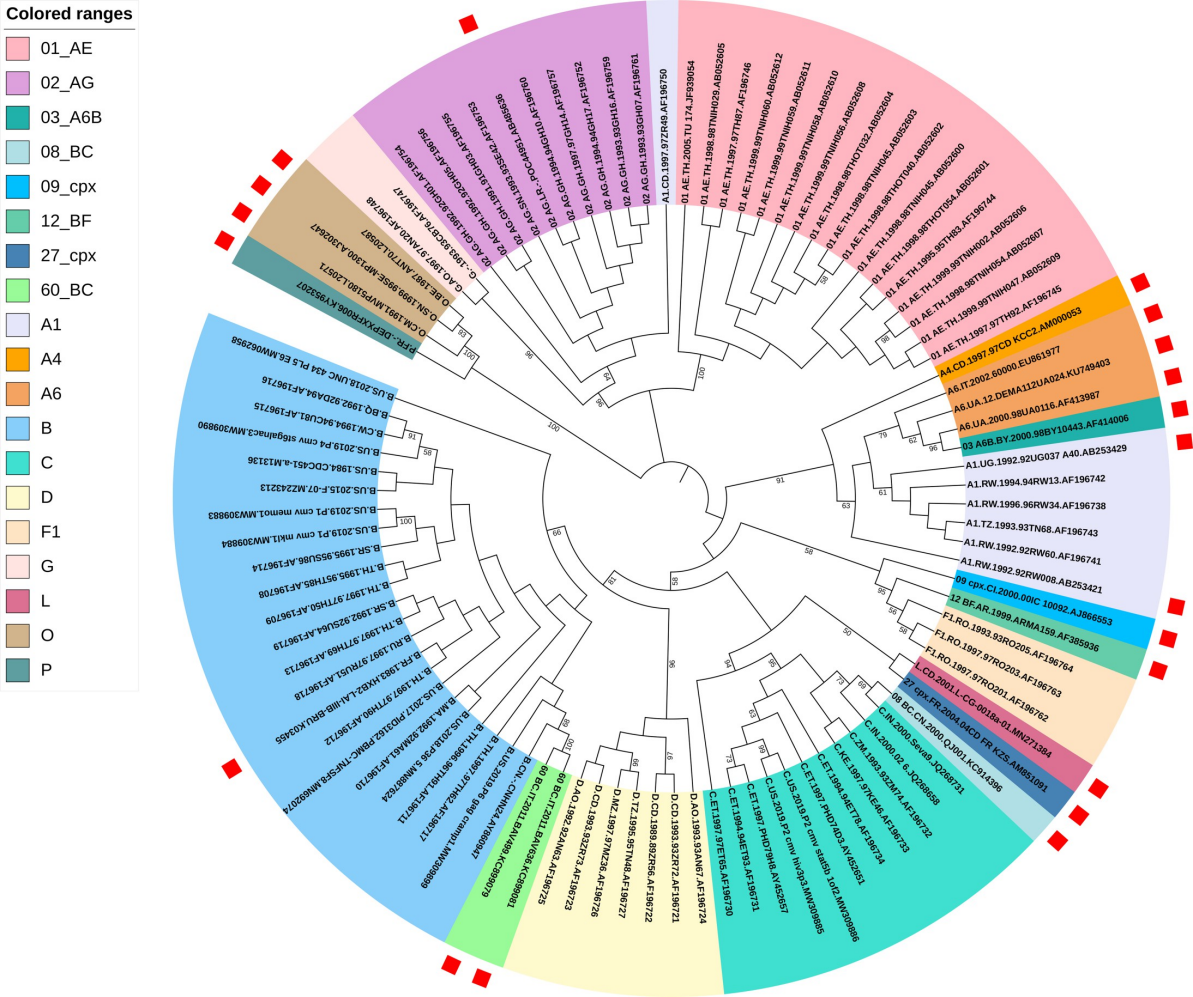
The ML phylogenetic tree built using the screened 76 solo 5’ LTRs sequences. The phylogenetic tree clearly showed confirmed distinct subtypes clusters, all supported by very strong bootstrap values. The best-fitting model of nucleotide substitution was GTR+G+I by using MEGA X. Tree topologies were searched using subtree-pruning-and-regrafting level 3 (SPR level 3) and the initial tree was made automatically (Default-NJ/BioNJ). The confidence of each node in the phylogenetic tree was determined using the bootstrap test with 500 replicates and values below 50% are not shown. The red squares represent 20 references identified from gold-standard sequences.

The screened 76 solo 5’ LTRs were used to construct a maximum likelihood phylogenetic tree by using MEGA X to identify distinct clusters, together with all the 20 provisional 5’ LTR references identified from gold-standard sequences. The best-fitting model of nucleotide substitution was generated and calculated as the general time reversible model with a gamma distribution and invariant sites (GTR+G+I) by using MEGA X. The sequence (accession number: AF196750) was first eliminated for subsequent classification because it is assigned as sub-subtype A1 while according to the phylogenetic tree it is displayed as CRF02_ AG in the HIV sequence database. Besides, the phylogenetic tree clearly showed confirmed distinct subtype clusters of all sequences including provisional 5’ LTR references from gold-standard sequences (Fig 2).

Considering countries and time distribution as well as the position relative to gold-standard sequences, 22 solo 5’ LTRs were assigned as the corresponding subtype references separately (S3 Table and S5 Dataset), which were classified into 4 subtypes, 2 sub-subtypes, and 2 CRFs (S3 Table).

### Reference classification based on 5’ LTRs with complete genomes

**Fig 3:**
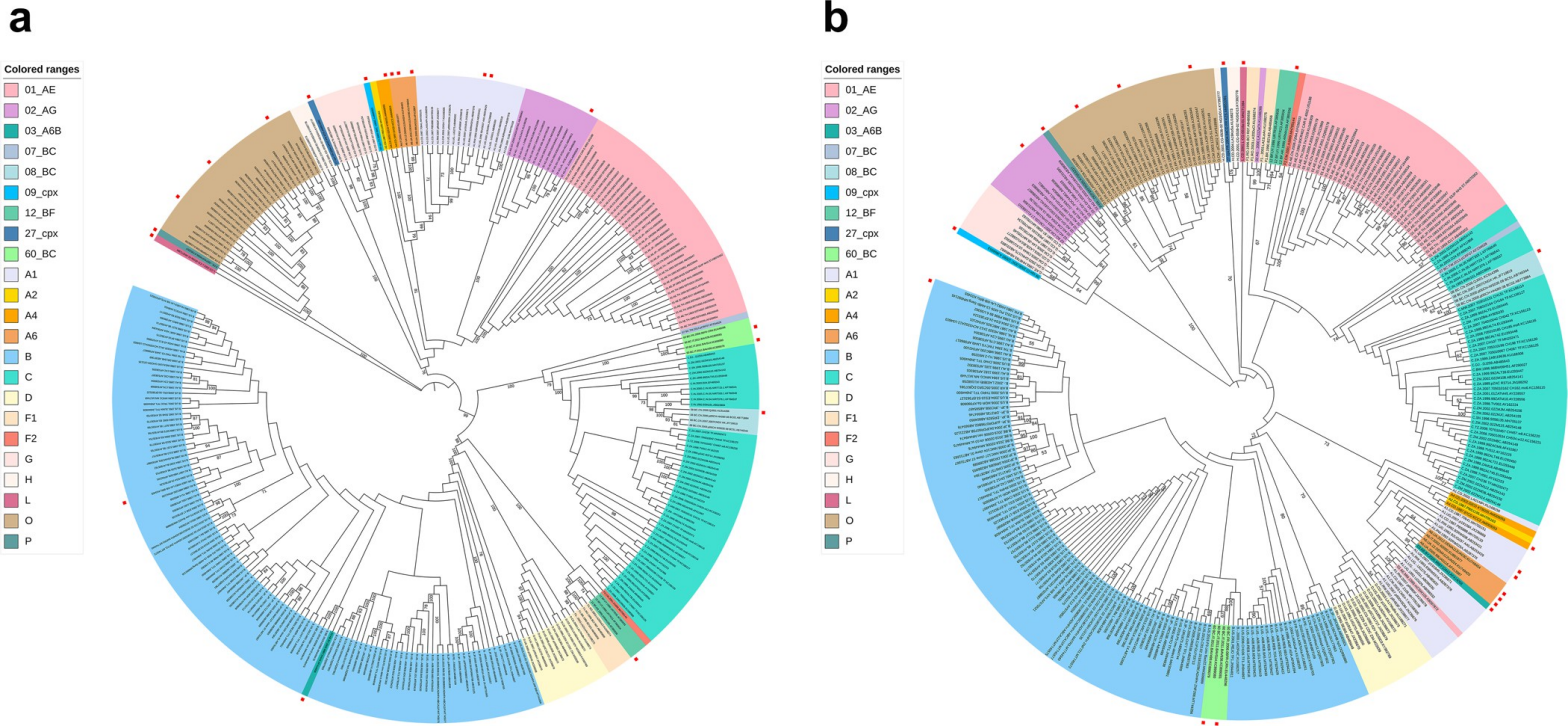
The ML phylogenetic analysis using 302 full-length genomes and 5’ LTRs extracted from them, respectively. (a) The maximum likelihood phylogenetic analysis by MEGA X based on 302 full-length sequences containing 5’ LTRs sequences. Sequences clustered in subtypes-specific branches with very high bootstrap values. (b) The maximum likelihood phylogenetic analysis by MEGA X based on extracted 5’ LTRs derived from the 302 full-length sequences. The confidence of each node in phylogenetic trees was determined using the bootstrap test with 500 replicates and values below 50% are not shown. Colored ranges represent different groups, subtypes, sub-subtypes, and CRFs. The red squares represent 20 provisional references identified from gold-standard sequences.

A total of 302 5’ LTRs sequences with complete genome were obtained after screening. Here, these full-length sequences together with all 20 provisional 5’ LTRs references carrying complete genome from gold-standard sequences were used to perform a maximum likelihood phylogenetic analysis using MEGA X. The best-fitting model of nucleotide substitution was generated and calculated as GTR+G+I by MEGA X. The results showed that sequences clustered in subtypes-specific branches with very high bootstrap values (Fig 3A). Subsequently, 5’ LTRs sequences were extracted from the complete genome sequences to perform another round of maximum likelihood phylogenetic analysis to verify the clustering (Fig 3B). The best-fitting model of nucleotide substitution was generated and calculated as GTR+G+I using MEGA X. The two phylogenetic trees were compared and sequences with inconsistent clustering were excluded. As shown in Fig 3A and 3B, the sequences belonging to subtype B, C, D, G, H, F1, CRF07_BC, CRF08_BC, CRF12_BF, group O, and P consistently clustered in both trees. Thus, these sequences are all preserved.

All 4 sequences of CRF60_BC, including 2 from gold-standard representatives (accession number: KC899079 and KC899081), formed a distinct cluster on each tree, with strong bootstrap values (Fig 3A and 3B). However, the two clusters displayed inconsistent topology. They nested within the subtype C clusters at the full-length analysis but nested within subtype B at the 5’ LTR level. We found that the reason is due to the fact that these 5’ LTRs sequences have been previously characterized to contain a large fragment spanning from 0 to 462bp, which phylogenetically belonged to subtype B [25]. Thus, these four sequences have also been preserved. Similarly, CRF03_A6B (accession number: AF414006) also formed a distinct cluster on each tree but displayed an inconsistent topology (Fig 3A and 3B). It nested within the subtype B clusters at the full-genome analysis but nested within subtype A6 at the 5’ LTR level. The 5’ LTR has been previously characterized to be A6 [54]. Thus, this CRF03_A6B has also been preserved. In addition, according to the definition of the recombinant structure by Vidal et al. [26], the CRF27_cpx (accession number: AM851091, the gold-standard sequence) with inconsistent topology between two trees was thus preserved.

Moreover, the phylogenetic analysis showed that sub-subtype A1, A2, A4, and A6 displayed a certain degree of topology dissociation. Considering the higher intra-subtypes sequence homology than the inter-subtypes sequence homology and that the dissociation occurred just within the whole cluster A, they were preserved as well.

It should be noted that there is the discrepancy of subtype L (accession number: MN271384, gold-standard sequence), sub-subtype F2 (accession number: MH705144), and CRF09_cpx (accession number: AJ866553, gold-standard sequence) and since no more than one sequence for each was available, they were all preserved but they were indicated with the asterisk (*), indicating risk warning as shown in Table 1.

**Table 1:** 5’ LTR reference sequence statistics.

Group/Subtype/Sub-subtype/CRF	5’ LTR references derived from Subtype Reference Alignmentsa	5’ LTR references based on solo sequences	5’ LTR references based on 5’ LTR sequences with complete genome	Consensus	Total
A1	A1.RW.1992.92RW008.AB253421	A1.RW.1992.92RW60.AF196741	A1.RW.2007.pR880F.JX236678	Consensus_A1	11
	A1.UG.1992.92UG037_A40.AB253429	A1.RW.1994.94RW13.AF196742	A1.UG.-.UG031.AB098330		
		A1.RW.1996.96RW34.AF196738	A1.UG.2007.p9004SDM.JX236676		
		A1.TZ.1993.93TN68.AF196743	A1.CD.2002.LA01AlPr.KU168256		
A2			A2.CD.1987.PBS1195.MH705163		1
A4	A4.CD.1997.97CD_KCC2.AM000053				1
A6	A6.IT.2002.60000.EU861977		A6.UA.2012.DEMA112UA030.KU749404	Consensus_A6	5
	A6.UA.2000.98UA0116.AF413987				
	A6.UA.2012.DEMA112UA024.KU749403				
B	B.FR.1983.HXB2-LAI-IIIB-BRU.K03455	B.TH.1997.97^TH^90.AF196712	B.US.1983.5084_83.AY835754	Consensus_B	8
		B.US.2018.UNC_434_PL5_E6.MW062958	B.JP.-.JRC05B.AB565497		
			B.US.1984.NY5CG.M38431		
			B.US.1983.5018_83.AY835777		
C		C.ET.1994.94ET78.AF196734	C.DJ.-.DJ259.AB485643	Consensus_C	7
		C.ET.1997.97ET65.AF196730	C.ZA.2007.705010198_CH198_TF.KC156130		
		C.US.2019.P2_cmv_hiv3p3.MW309885	C.IN.1993.93IN101.AB023804		
D		D.AO.1993.93AN67.AF196724	D.UG.2008.p191859.JX236672	Consensus_D	7
		D.CD.1989.89ZR56.AF196722	D.CD.1985.Z2Z6_Z2_CDC_Z34.M22639		
		D.TZ.1995.95TN48.AF196727	D.CD.2003.LA17MuBo.KU168271		
G		G.-.1993.93CB76.AF196747	G.KE.1993.HH8793.AB485662	Consensus_G	5
		G.AO.1997.97AN20.AF196748	G.CD.1987.87-2580.MH705162		
F1		F1.RO.1993.93RO205.AF196764	F1.-.2003.LA21LeAn.KU168275	Consensus_F1	6
		F1.RO.1997.97RO201.AF196762	F1.RO.1996.BCI_R07.AB485658		
			F1.BR.1990.BZ163.AB485656		
F2			F2.CM.2001.A1699.MH705144^*^		1
H			H.CD.2001.CG-0536-02_NGSID14.KY392777	Consensus_G	4
			H.CD.2001.CG-0538-02_NGSID15.KY392778		
			H.CD.2004.LA19KoSa.KU168273		
L	L.CD.2001.L-CG-0018a-01.MN271384^*^				1
CRF01_AE		01_AE.TH.1995.95^TH^83.AF196744	01_AE.TH.1993.93TH054.AB220945	Consensus_CRF01_AE	8
		01_AE.TH.1997.97^TH^92.AF196745	01_AE.JP.1993.93JP_NH1.AB052995		
		01_AE.TH.1998.98TNIH045.AB052603	01_AE.CN.2005.FJ053.DQ859179		
		01_AE.TH.2005.TU_174.JF939054			
CRF02_AG	02_AG.LR.-.POC44951.AB485636	02_AG.GH.1994.94GH17.AF196752	02_AG.FR.-.DJ263.AB485634	Consensus_CRF02_AG	6
		02_AG.SN.1993.93SE42.AF196753	02_AG.CM.2007.CM100-17.KU168310		
CRF03_A6B	03_A6B.BY.2000.98BY10443.AF414006				1
CRF07_BC			07_BC.TW.2013.pCRF07.KF234628		1
CRF08_BC	08_BC.CN.2000.QJ001.KC914396		08_BC.CN.2000.p00CH-HH090_08_BC02.AB773884	Consensus_CRF08_BC	4
			08_BC.CN.2007.2007CNGX_HK.JF719819		
CRF09_cpx	09_cpx.CI.2000.00IC_10092.AJ866553^*^				1
CRF12_BF	12_BF.AR.1999.ARMA159.AF385936		12_BF.UY.1999.URTR23.AF385934	Consensus_CRF12_BF	4
			12_BF.UY.1999.URTR35.AF385935		
CRF27_cpx	27_cpx.FR.2004.04CD_FR_KZS.AM851091				1
CRF60_BC	60_BC.IT.2011.BAV499.KC899079		60_BC.FR.2006.06FR-CRN.EU448296	Consensus_CRF60_BC	5
	60_BC.IT.2011.BAV636.KC899081		60_BC.IT.2011.BAV514.KC899080		
O	O_BE_87_ANT70_L20587		O.CM.1995.LA29YBF26.KU168281	Consensus_O	8
	O_CM_91_MVP5180_L20571		O.CM.1999.AB260.MH705147		
	O_SN_99_99SE_MP1300_AJ302647		O.FR.2001.LA46BCF03.KU168289		
			O.FR.1998.LA30RBF125.KU168282		
P	P_FR_x_DEPXXFR006_KY953207				1
Total	20	22	41	14	97

^a^ Subtype Reference Alignments are provided in the HIV sequence database (https://www.hiv.lanl.gov/content/sequence/HIV/mainpage.html). For each subtype, 4 genomes were selected as being broadly representative of that subtype. For CRFs included, with up to 4 genomes provided for each.

^*^ indicates risk warning.

From the comparison of the two phylogenetic trees conducted in this study, we found that one sequence (accession number: AB097872) nested within CRF01_ AE cluster at the full-genome level, but the 5’ LTR clustered with A1, in contrast with other CRF01_AE sequences. The remaining sequences all consistently clustered in the same manner in both trees. Thus, this sequence (accession number: AB097872) was excluded. In order to investigate the inconsistency between the 5’ LTR and the internal region subtype of the AB097872, we retrieved the 3’ LTR of AB097872 (547bp). Through phylogenetic analysis and comparison, it was found that the 5’ LTR is nested within A1 cluster, and the 3’ LTR is nested within CRF01_ AE cluster. The results indicated that this is probably just a sequencing error and confirm the accuracy of exclusion. Besides, one sequence belonging to CRF02_AG (accession number: KU168266) clustered within CRF02_AG in the complete genome-based analysis, as expected. However, its 5’ LTR region clustered to F1 subtype. This sequence was also removed

Considering countries and time distribution as well as the position relative to gold-standard sequences, a total of 43 5’ LTR sequences with complete genome were assigned as the corresponding subtype references separately (S3 Table), which were classified into 1 group (O), 5 subtypes, 6 sub-subtypes, and 6 CRFs (S3 Table).

### Definitive determination of reference strains and genetic analysis of HIV-1 5’ LTRs sequences

One of the 4 recognized principles for the HIV-1 subtype classification is the absence of intersubtype recombination [22, 35, 50]. Thus, the tool of detecting recombination, RDP4 [38], was applied to investigate whether any recombination occurred among the 85 5’ LTR references sequences. It was found that a sequence of subtype G (accession number: MH705145) and a sequence belonging to subtype A4 (accession number: AM000055) are recombinants. The sequence of subtype G (accession number: MH705145) is a recombinant result from unknown (major parent) and A1 (accession number: AF196741, minor parent) with the recombination signal supported by 7 recombination methods (RDP, GENECONV, BootScan, Maxchi, Chimaera, SiSscan, and 3Seq). The sequence of sub-subtype A4 (accession number: AM000055) was a recombinant from A1 (major parent, accession number: AB098330) and an unknown sequence (minor parent) with the recombination signal supported by 5 recombination methods used (RDP, BootScan, MaxChi, SiSscan, and 3Seq). Based on the 4^th^ principle of the reference system construction [22, 35], these two sequences were removed from the classified reference sequences evaluated in this study. It should be noted that even if one of the genome used by Vidal et al [55] to establish the nomenclature of the A4 sub-subtype is recombinant, this does not necessarily invalidate that A4 classification. Like other subtype and sub-subtype classification, A4 classification is of great benefit to the field.

**Fig 4:**
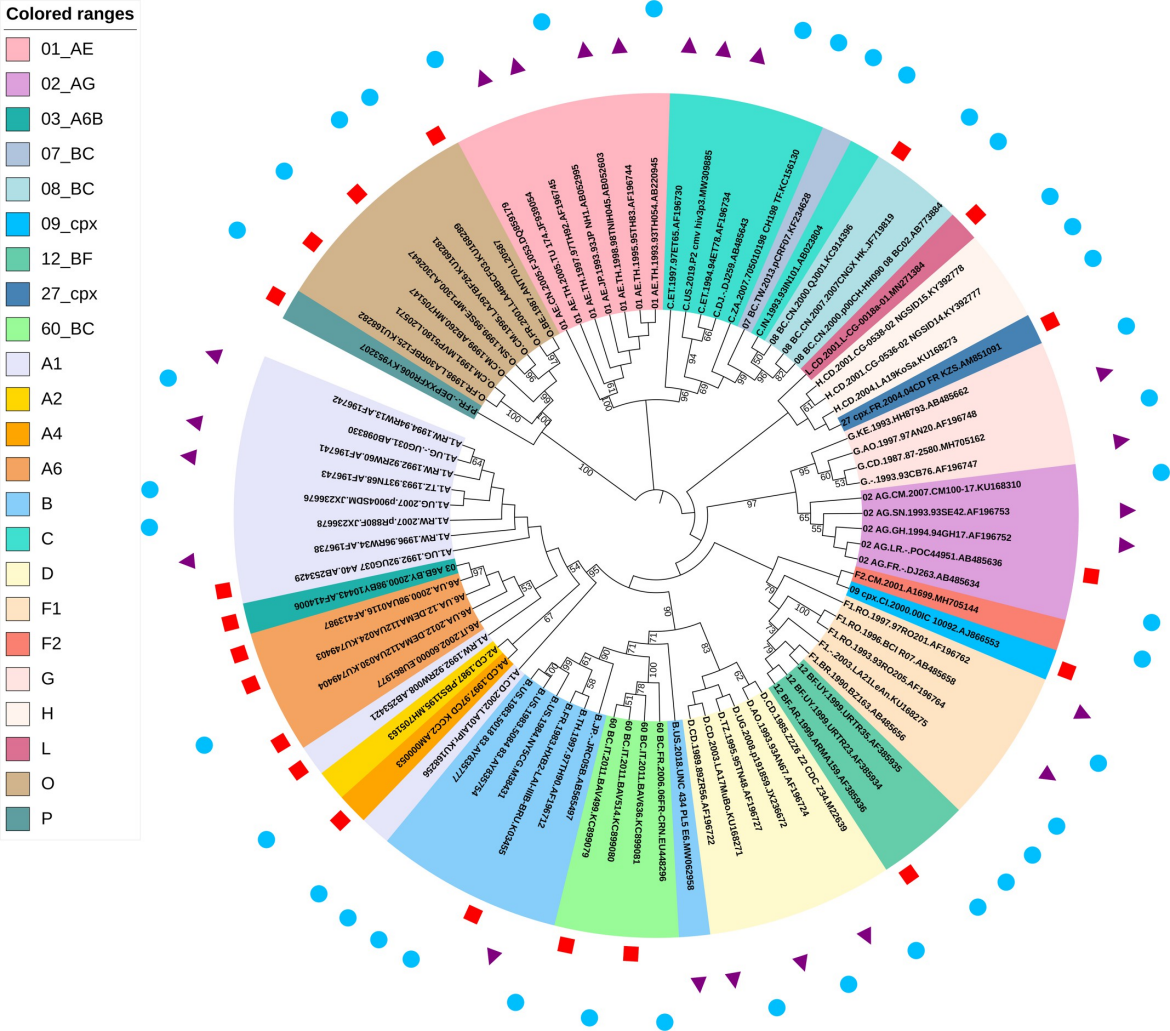
The phylogenetic tree of 83 definitive screened reference representatives of HIV-1 5’ LTRs. Representatives of each confirmed group, subtype, sub-subtype, and CRF were aligned and a phylogenetic tree was constructed using the maximum likelihood method by MEGA X. The results indicated that major distinct clusters are all with reliable bootstrap support and values below 50% are not shown (replicates = 500). Tips are labeled by groups/subtypes/sub-subtypes/CRFs, sample country, sample year, sequence name, and accession number. Colored ranges represent different groups, subtypes, sub-subtypes, and CRFs. The red squares represent 20 references identified from gold-standard sequences. The purple triangles represent 22 references identified from solo 5’ LTRs. The blue dots represent 41 references identified from 5’ LTR sequences with complete genome.

Therefore, a total of 83 subtype references covering 2 groups, 6 subtypes, 6 sub-subtypes, and 9 CRFs were selected as reference representatives (Table 1 and S6 Dataset). Then, these representative sequences were aligned and used for constructing a phylogenetic tree. Despite the length of the 5’ LTR region is short (634 bp) and the nucleotide identity is much higher than that of HIV-1 internal region, distinct clusters were still evident, even within sub-subtypes (Fig 4). There is only one representative reference for subtype A2, A4, F2, L, CRF03_A6B, CRF07_BC, CRF09_cpx, CRF27_cpx, and group P. For subtype H, there formed a separate cluster but with low bootstrap support. On the contrary, all other clusters showed high bootstrap support. Further, 14 consensus sequences of 5’ LTR references were also constructed (Table 1 and S7 Dataset).

**Fig 5:**
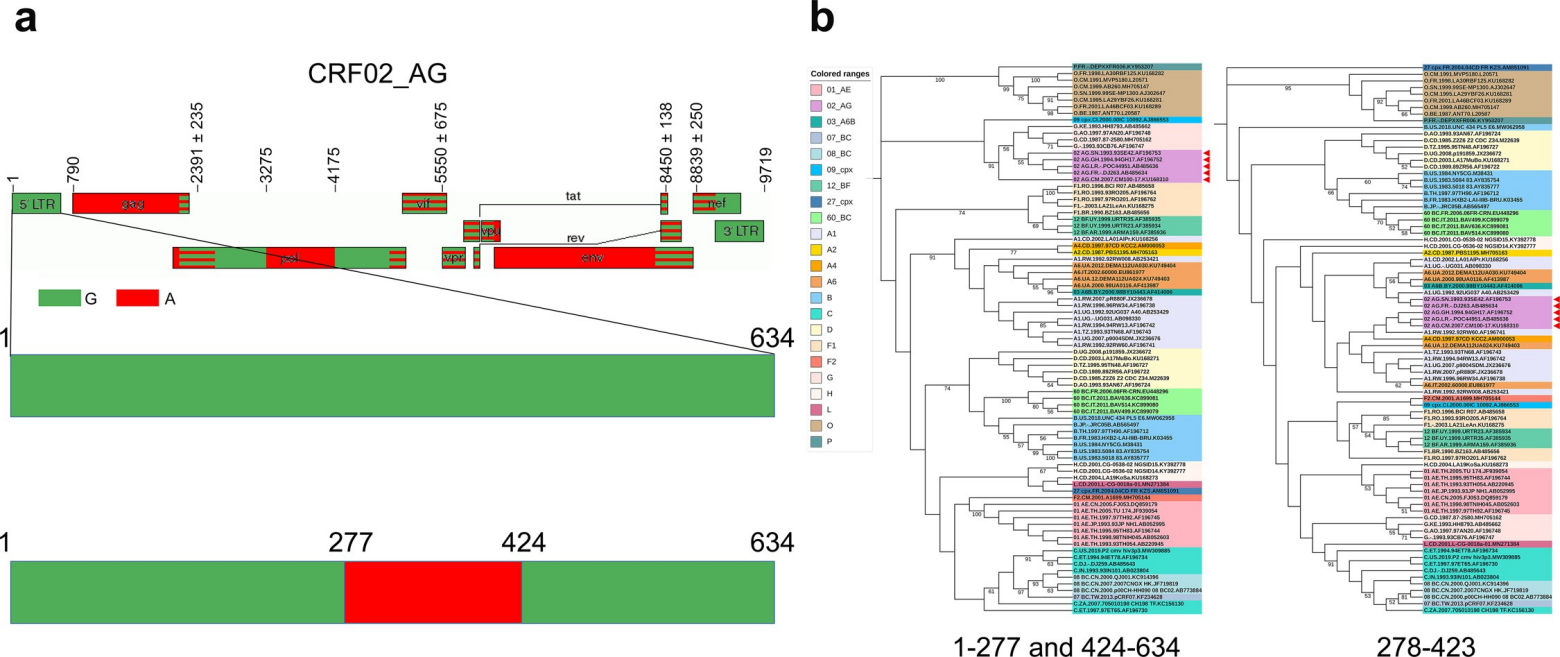
The recombination characterization of 5’ LTR of CRF02_AG references. The recombination analysis and phylogenetic analysis confirm that the 5’ LTRs of all 5 CRF02_AG representatives are recombinants with the same mosaic manner (including 02_AG.LR.-.POC44951.AB485636 from gold standard representatives in *Subtype Reference Alignments*). (a) Updated genome maps of CRF02_AG 5’ LTR. The top picture shows the original mosaic structure from the Los Alamos HIV sequence database. The below picture displays the updated recombinant details of 5’ LTR. The standard representatives are marked by different clors, as indicated. (b) The phylogenetic confirmation of the recombination pattern by sub-region trees using extracted fragments. The left picture shows the phylogenetic relationship of the region spanning HXB2 nt 1–277 and 424-634bp with all classified 5’ LTRs representatives in the current work. The right picture shows the phylogenetic relationship of the region spanning HXB2 nt 278-423bp with all classified 5’ LTRs representatives in the current work. The tree was constructed using the Maximum likelihood method implemented in MEGA X. The values at the nodes indicate the percent bootstraps in which the cluster to the right was supported. Colored ranges represent different groups, subtypes, sub-subtypes, and CRFs. The red triangles represent recombinants.

It is worth to be noted that recombination exclusion analysis showed that the 5’ LTRs sequences belonging to 5 CRF02_AG representatives are all recombinants with the same mosaic manner (Fig 5A). Specifically, the 5’ LTR sequence of sub-subtype A1 was inserted into the skeleton of the 5’ LTR sequence of subtype G at the position of 278-423bp (position according to the K03455 coordinates) (Fig 5A). To further confirm the recombination analysis results, the fragments of parental strains were extracted and sub-region phylogenetic trees were constructed with parental strains using MEGA X. The results showed that the major parental strains belonged to subtype G with bootstrap support values of 91% (Fig 5B), while the minor parental strains clustered within sub-subtype A1 (Fig 5B). The length of the segment was only 146 bases, and as discussed by Leitner et al. [56], region length is not sufficient to produce phylogenetic trees with high bootstrap values, and similarly to previously reported, a high bootstrap value was not obtained in this study either, despite a distinct cluster still obtained. Therefore, it was clearly demonstrated in this study that the 5’ LTR sequences belonging to CRF02_AG subtypes were all recombinants.

### Reliability test of newly established 5’ LTR classification system

To test the reliability and applicability of our established classification system, the constructed references were first applied to identify the 5’ LTR assignment of the clinical isolates in China. Genes of *gag*, *pol*, and *env* per isolate were amplified by RT-PCR, sequenced, and assembled as described in previous works [57–59]. In addition, the two partial LTRs sequences at the 5’ terminus and 3’ terminus were also amplified, sequenced, assembled, and further combined via the R region to obtain a complete LTR sequence, as previously described [6]. Thus, a total of 22 sequences including 4 regions (LTR, *gag*, *pol*, and *env*) were obtained.

**Fig 6:**
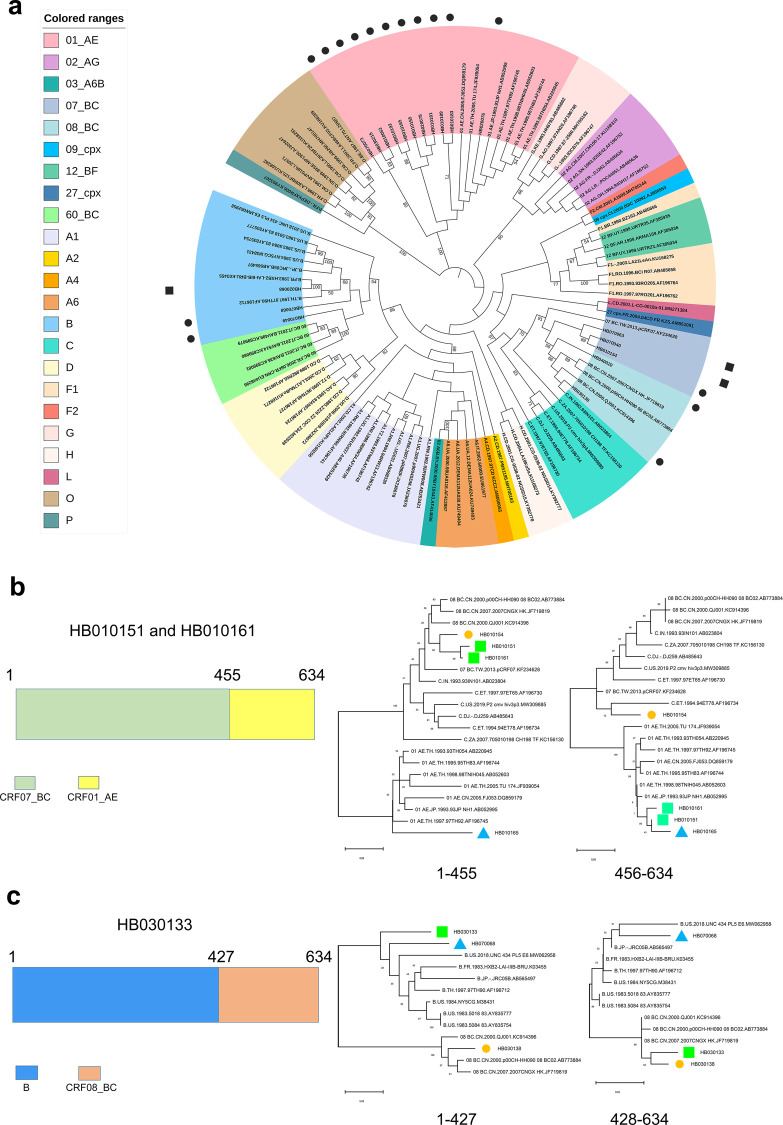
The reliability test of newly established 5’ LTR classification system by identifying the 5’ LTR assignment of the clinical isolates in China. (a) The ML phylogenetic tree was built using 19 amplified HIV-1 5’ LTR sequences together with 83 HIV-1 5’ LTR references. These 19 5’ LTR contained no recombination and showed good clustering with strong bootstrap support. The black dot represents 16 5’ LTRs having consistent subtype assignment with that of the 3 other regions including *gag*, *pol*, and *env*. The black square represents 3 5’ LTRs having inconsistent subtype assignments with that of the 3 other regions. Colored ranges represent different groups, subtypes, sub-subtypes, and CRFs. (b) The same recombination pattern of the 2 5’ LTR recombinants (HB010151 and HB010161) and the subsequent confirmation by sub-region trees. The 2 recombinants share a common mosaic pattern. The sub-region phylogenetic analysis confirmed that the extracted segments have the right subtype assignment as indicated by recombination analysis. The green square represents recombinants. The blue triangle represents major parent. The yellow dot represents minor parent. (c) The recombination pattern of HB030133 and the subsequent confirmation by sub-region trees. The sub-region phylogenetic analysis confirmed that the extracted segments have the right subtype assignment as indicated by recombination analysis. The green square represents recombinant. The blue triangle represents major parent. The yellow dot represents minor parent.

Subsequently, a subtyping test of these 22 sequences by using the 3 regions (*gag*, *pol*, and *env*) was performed as previously described [57–59], followed by a subtyping test based on the 5’ LTRs which was performed using the classification system developed in this study. The results showed that 5’ LTRs of 16 out of 22 displayed consistent subtype assignment with the subtype assignment by using 3 other regions (*gag*, *pol*, and *env*). These subtypes included 2 subtype B, 8 CRF01_AE, 1 CRF07_BC, 2 CRF08_BC, and 3 CRF55_01B (Table 2 and Fig 6A). Unexpectedly, 3 sequences showed different subtype assignments in the 5’ LTRs compared to that obtained with the other 3 regions. HB020068 was demonstrated to belong to subtype CRF07_BC based on *gag*, *pol*, and *env* regions, but it was classified as subtype B based on the 5’ LTR region (100% bootstrap support). On the contrary, HB070040 and HB070063 belonged to subtype B based on *gag*, *pol*, and *env* regions, but to CRF07_BC based on 5’ LTR regions (Table 2 and Fig 6A). The results revealed that the whole 5’ LTR of all 3 sequences was exchanged via genetic recombination. Furthermore, the other 3 sequences showed an exchange of a certain proportion of fragments within the 5’ LTR via recombination, including HB010151, HB010161, and HB030133 (*p*-value≤0.05). The recombinant HB010151 was produced by the recombination between HB010154 (CRF07_BC) and HB010165 (CRF01_AE). A segment of 455 bp of CRF07_ BC was inserted at positions 1–455 (position according to the K03455 coordinates) in the skeleton of CRF01_AE (*p*-value≤0.05). HB010161 sequence displayed the same recombination events as HB010151 (Fig 6B). The recombinant HB030133 resulted from the recombination between HB070068 (subtype B) and HB030138 (CRF08_BC). A segment of 207 bp of CRF08_ BC was inserted at positions 428–634 (position according to the K03455 coordinates) within the skeleton of subtype B (*p*-value≤0.05, Fig 6C). The mosaic structures were all confirmed by sub-region phylogenetic trees by using the fragments extraction (Fig 6B and 6C). These results validated the accuracy of the classification system and its practical applicability and demonstrated that the whole classification system could facilitate the characterization of HIV-1 5’ LTRs.

**Table 2:** Assignments of 4 regions of the 22 amplified samples.

Sample	Assignment of *gag*	Assignment of *pol*	Assignment of *env*	Assignment of LTR
HB070049	B	B	B	B
HB070068	B	B	B	B
HB010155	CRF01_AE	CRF01_AE	CRF01_AE	CRF01_AE
HB010165	CRF01_AE	CRF01_AE	CRF01_AE	CRF01_AE
HB010181	CRF01_AE	CRF01_AE	CRF01_AE	CRF01_AE
HB010183	CRF01_AE	CRF01_AE	CRF01_AE	CRF01_AE
HB010189	CRF01_AE	CRF01_AE	CRF01_AE	CRF01_AE
HB010192	CRF01_AE	CRF01_AE	CRF01_AE	CRF01_AE
HB020076	CRF01_AE	CRF01_AE	CRF01_AE	CRF01_AE
HB020078	CRF01_AE	CRF01_AE	CRF01_AE	CRF01_AE
HB010154	CRF07_BC	CRF07_BC	CRF07_BC	CRF07_BC
HB030138	CRF08_BC	CRF08_BC	CRF08_BC	CRF08_BC
HB040020	CRF08_BC	CRF08_BC	CRF08_BC	CRF08_BC
HB030215	CRF55_01B	CRF55_01B	CRF55_01B	CRF55_01B
HB070073	CRF55_01B	CRF55_01B	CRF55_01B	CRF55_01B
HB010053	CRF55_01B	CRF55_01B	CRF55_01B	CRF55_01B
HB020068	CRF07_BC	CRF07_BC	CRF07_BC	B
HB070040	B	B	B	CRF07_BC
HB070063	B	B	B	CRF07_BC
HB010151	CRF01_AE	CRF01_AE	CRF01_AE	CRF07_BC&CRF01_AE
HB010161	CRF01_AE	CRF01_AE	CRF01_AE	CRF07_BC&CRF01_AE
HB030133	B	B	B	B&CRF08_BC

### Recombination in 5’ LTRs caused a significant change of transcription factor binding sites

We further explored the TFBS differences within 5’ LTRs of 6 identified recombinants circulating in China, including HB020068, HB070040, HB070063, HB010151, HB010161, and HB030133. The results revealed that recombination introduces extensive changes of a large number of TFBSs. For the exchange of 5’ LTR between subtype B and CRF07_BC (HB020068, HB070040, and HB070063), the exchange via recombination leads to a change of 1011 TFBSs (S4 Table). For the exchange of partial 5’ LTR between CRF01_AE and CRF07_BC (HB010151 and HB010161, Fig 6B), the exchange via recombination leads to a change of 1326 TFBSs (S5 Table). For the exchange of partial 5’ LTR between subtype B and CRF08_BC (HB030133, Fig 6C), the exchange via recombination leads to a change of 244 TFBSs (S6 Table). These results suggested the recombination events may cause the change of binding of hundreds of transcription factors at 5’ LTR.

**Fig 7:**
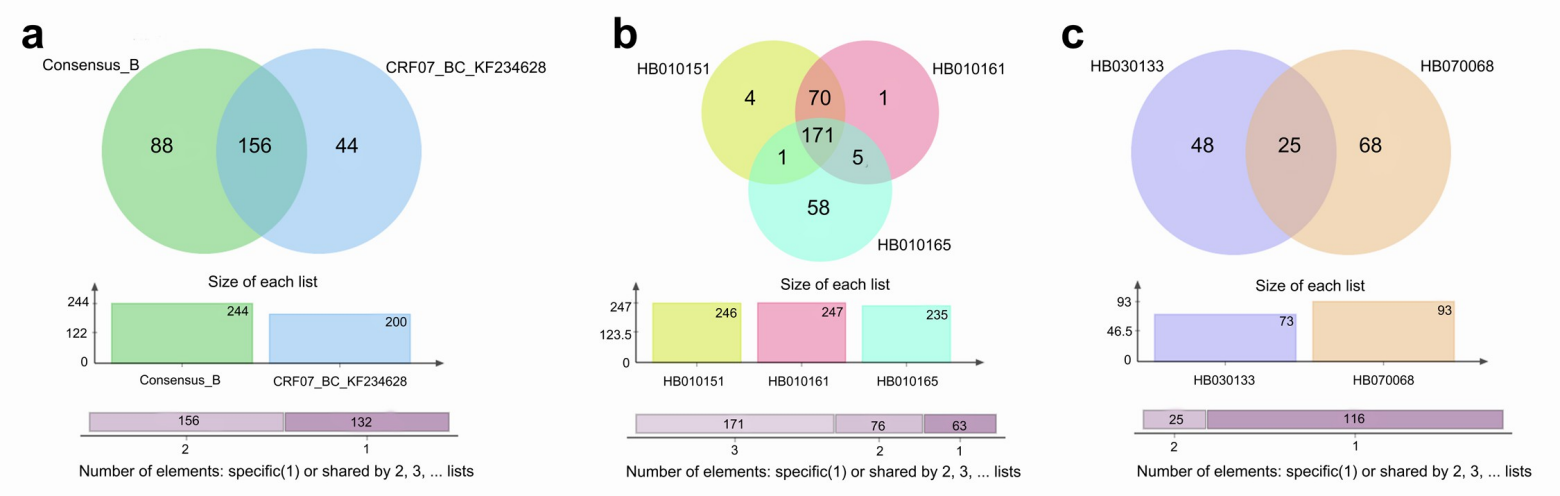
Venn diagram representation of the significant changes of transcription factors caused by recombination at 5’LTR. (a) Change of transcription factors via recombination between the CRF07_BC and the subtype B at 5’ LTR (the recombinant HB020068, HB070040, and HB070063). The green indicates specific transcription factors of subtype B. The blue indicates specific transcription factors of CRF07_BC. The overlap indicates transcription factors shared by subtype B and CRF07_BC. (b) Change of transcription factors via recombination between the recombinant HB010151, HB010161, and the parent strain HB010165 at 5’ LTR. The yellow indicates specific transcription factors of HB010151. The pink indicates specific transcription factors of HB010161. The cyan indicates specific transcription factors of HB010165. The overlap of yellow and pink indicates transcription factors shared by HB010151 and HB010161. The overlap of pink and cyan indicates transcription factors shared by HB010161 and HB010165. The overlap of yellow and cyan indicates transcription factors shared by HB010151 and HB010165. The overlap of yellow, pink, and cyan indicates transcription factors shared by HB010151, HB010161, and HB010165. (c) Change of transcription factors via recombination between the recombinant HB030133 and its parent strain HB070068 at 5’ LTR. The purple indicates specific transcription factors of HB030133. The wheaten indicates specific transcription factors of HB070068. The overlap indicates transcription factors shared by HB030133 and HB070068.

Then, the transcriptional factors bound at 5’ LTR before and after recombination was further analyzed by comparison of the parent strain with the progeny recombinant. Specially, we found that although there were 156 common proteins before and after recombination between the CRF07_BC and the subtype B (the recombinant HB020068, HB070040, and HB070063), these proteins showed different binding position within 5’ LTR after the recombination (Fig 7A and S4 Table). Regarding to the other 3 recombinants, HB010151 and HB010161 was separately compared with the parent strain HB010165. As expected, the transcriptional factors bound at 5’LTR was quite similar between the two recombinant, HB010151 and HB010161. However, the association of 58 transcriptional factors with 5’ LTR were completely diminished after the recombination in both HB0151 and HB010161 (Fig 7B and S5 Table), in comparison to the parent strain HB010165. Besides, there were only 25 common proteins associated with 5’LTR between the recombinant HB030133 and its parent strain HB070068. However, the location of these 25 proteins within 5’LTR were also changed after the recombination (Fig 7C and S6 Table). Among these binding proteins at 5’LTR after recombination, some are very important transcription factors such as AP-1, p53, SP1, P300, and NF-κB (S4–S6 Tables). Together, these data further demonstrated the usefulness of this newly constructed 5’LTR classification system, and highlighted the importance of 5’ LTR characterization in the study of HIV-1 classification, evolution, and biological characterization.

## Discussion

The classification of a virus is rather critical, having a significant impact on molecular epidemiology, basic mechanism, and clinical science. The 5’ LTR accounts for only 6.52% of the full-length HIV-1 genomic RNA but plays crucial roles in the whole life cycle, including the reverse transcription, integration, and transcriptional regulation of HIV-1. In particular, the HIV-1 5’ LTR possesses promoters and enhancers that can be used to drive virtual transcription, and the biological differences of 5’ LTR of some different strains have been described [18, 24]. However, the research on 5’ LTR lags far behind that on internal genes, such as *gag*, *pol*, and *env*, which was restricted by the fact that a systematic phylogenetic classification system of HIV-1 5’ LTR has never been established, although some studies have performed indirect LTR characterization by using the internal gene classification [2, 17–19, 24]. This may underestimate the effects of LTR diversity on HIV-1 characterization and evolution.

All remaining regions of the HIV-1 genome have been systematically and phylogenetically classified and the classification has been widely used, except for LTR [22, 60–63]. However, the rigid need to partition the LTR systematically and phylogenetically has always existed. When performing the comparison of biological activity of LTRs of different subtypes or when performing the identification and characterization of clinically isolated LTRs, as what is done with other areas like *gag*, *pol*, *env*, or other 6 function regions, researchers in the field even do not know what the exact representative of LTR of each subtype is [2, 17–19, 24]. The well-known posterior probabilities-based HIV-1 recombination detection tool, jpHMM, is unable to provide accurate subtyping information of the 5’ LTR region of a query due to the absence of 5’ LTR classification [64]. Besides, up to August 17, 2021, there were 835940 entries with classified subtype information in the HIV sequence database. Among them, there were only 722 entries of 5’ LTR, accounting for 0.086%. Some classified subtypes/CRFs have definite 5’ LTR representatives. However, 5’ LTR regions of more subtypes and CRFs are still uncertain. Usually, the phylogenetic classification of internal regions was also used to characterize 5’ LTRs expediently. For example, when an internal region of a strain was identified by the phylogenetic classification of internal regions as a specific subtype, the 5’ LTR of the stain was considered to be the same subtype automatically [2, 17–19, 24]. This is rather lax. For a virus with particularly obvious recombination characteristics and for a region with 634 bp, the reference sequences corresponding to this region were not used for characterization and the characterization result of the internal region was directly applied to LTR. This is not rigorous. With the increasing recognition of the important role played by 5’ LTR, it is urgent to supplement a systematic classification of LTR regions. Thus, following exactly the same methods and principles cited by the phylogenetic classification of internal regions [22, 35], this study has supplemented a unified and comprehensive classification of HIV-1 5’ LTRs and explored the effect of classification-based 5’ LTR genetic variation on the evolution and biological characteristics of HIV-1. To ensure the reference construction, the 4 recognized formal rules for the subtype nomenclature system were strictly followed. By applying the classification system to 22 Chinese epidemic strains, the classification has been proven practical applicability, revealing some interesting results unclear previously. Specifically, we found the occurrence of recombination within 5’ LTR of these strains, which has caused the binding change of many important transcriptional factors and most likely would affect the transcriptional activity of 5’ LTR. In summary, we did not supplement a different classification system for LTRs of the genome. The issue is that the current widely used phylogenetic classification system of the internal regions for HIV-1 classification does not involve the HIV-1 LTR region. Following exactly the same methods and principles cited by the classification of internal regions [22], we just supplemented the phylogenetic classification of LTRs in the current work. This supplemented classification system will help to facilitate the understanding of the diversity characteristics and recombination rules of LTRs, improving our research on HIV-1 epidemiology and evolution.

The remarkable genetic variation and population plasticity is a prominent feature of HIV-1 [65]. This genetic variation originates from two sources, including point mutations and genetic recombination [6, 51]. It has been found that frequent genetic recombination existed within HIV-1 genome [6, 36, 37]. Our previous work updated the molecular model on HIV-1 genetic recombination [51], which has proposed that a large number of micro-recombination were considered as mutations because they cannot be detected due to the limitation of detection tool strategies and capabilities [51]. Compared with mutation, recombination has a much greater remodeling effect on genome structure, as it can lead to a large fragment exchange. For decades, recombinant analysis has revealed 134 CRFs and countless URFs. However, few studies have focused on the recombination analysis within 5’ LTR regions because of the lack of systematic reference sequences. This study has demonstrated that our classification system can effectively improve the characterization of HIV-1 5’ LTRs. In addition, it should be noted it is not that if a region can generate recombination, the region cannot be used as a classification marker. If so, the reference sequences of those internal areas cannot be used. The actual conclusion is that there must be the exclusion of recombination in the screened reference sequences as we have performed.

In recent years, CRF01_AE and CRF07_BC have been found as dominant HIV-1 strains in China. The discovery of new recombinants within the 5’ LTR region suggested that the prevalence of HIV-1 strains in China could result in the recombination of CRF01_AE and CRF07_BC subtypes. This may generate new recombinants carrying the parental advantages and developing into new epidemic strains. Meanwhile, it also reminded us that the transmission of HIV-1 in China tends to be more complex, while the prevention, control, and treatment of HIV-1 are facing great challenges.

A limitation of the present work is that there are still no classified references for all groups, subtypes, sub-subtypes, and CRFs. With the increase of HIV-1 5’ LTRs and complete sequences obtained, the reference classification system will be improved as the great enrichment of sequences in the HIV sequence database. As the increasing investigation on 5’ LTRs diversity and characterization, we will get a deeper understanding of HIV-1 transmission, evolution, and the basic mechanism of transcriptional regulation. Therefore, our study has proposed a comprehensive classification for HIV-1 5’ LTR, which will facilitate the research on molecular epidemiology, virus detection, and diagnosis, clinical treatment, drug and therapy development.

## Supporting information

S1 Table Primers used for amplification of the 5’ R-U5-NCR and 3’ U3-R regions of the HIV-1 genome. (XLSX)

S2 Table The details of excluded sequences. (XLSX)

S3 Table Reference sequence statistics midway. (XLSX)

S4 Table The differences of TFBS within 5’ LTR between subtype B and CRF07_BC via recombination. (XLSX)

S5 Table The differences of TFBS within 5’ LTR between CRF01_AE and CRF07_BC via recombination. (XLSX)

S6 Table The differences of TFBS within 5’ LTR between subtype B and CRF08_BC via recombination. (XLSX)

S1 Dataset 497 5’ LTR sequences of HIV-1 retrieved from HIV database (one sequence per patient). (TXT)

S2 Dataset 302 5’ LTRs with full-length sequences. (TXT)

S3 Dataset 76 solo 5’ LTR sequences. (TXT)

S4 Dataset 20 5’ LTRs with complete genome from gold-standard sequences. (TXT)

S5 Dataset 22 5’LTR references based on solo sequences. (TXT)

S6 Dataset Finally identified 83 references. (TXT)

S7 Dataset 14 consensus. (TXT)
